# Reversible Systolic Heart Failure in a Patient on Ibrutinib Chemotherapy

**DOI:** 10.7759/cureus.23266

**Published:** 2022-03-17

**Authors:** Misbahuddin Khaja, Hitesh Gurjar, Laura Yapor, Minu C Abraham, Nolberto Hernandez, Asim Haider

**Affiliations:** 1 Pulmonary and Critical Care, BronxCare Health System, Affiliated With Icahn School of Medicine at Mount Sinai, Bronx, USA; 2 Internal Medicine, BronxCare Health System, Affiliated With Icahn School of Medicine at Mount Sinai, Bronx, USA; 3 Internal Medicine, Bronxcare Health System, Affiliated With Icahn School of Medicine at Mount Sinai, Bronx, USA

**Keywords:** lymphoma, reduced ejection fraction, cardiac arrythmia, heart failure, cardiomyopathy, ibrutinib

## Abstract

Ibrutinib is an irreversible Bruton tyrosine kinase inhibitor that is approved for the treatment of mantle cell lymphoma, chronic lymphocytic leukemia, small lymphocytic lymphoma, Waldenström macroglobulinemia, marginal zone lymphoma, and mantle cell lymphoma. However, it is associated with significant cardiotoxic effects, with hypertension and atrial fibrillation being the most common. We present the case of a 42-year-old female with a medical history significant for lymphoplasmacytic lymphoma who presented with non-arrhythmic, non-ischemic cardiomyopathy after four months of chemotherapy with ibrutinib. In addition, her left ventricular ejection fraction improved markedly within a few days of stopping ibrutinib. We propose that the use of ibrutinib may be associated with reversible non-ischemic cardiomyopathy even in the absence of cardiac arrhythmias. Therefore, clinicians should be cognizant of the signs and symptoms of cardiomyopathy in patients on ibrutinib chemotherapy.

## Introduction

Ibrutinib is an antineoplastic drug and an irreversible blocker of Bruton tyrosine kinase (BTK). BTK plays an essential role in the maturation of B cells and subsequent proliferation of antibodies. Inhibition of BTK prevents tumor cell migration and adhesion [1]. Moreover, it induces apoptosis of tumor cells [2]. Ibrutinib was approved by the US Food and Drug Administration (FDA) in 2013 to treat B-cell malignancies [3]. It is currently approved to treat mantle cell lymphoma, chronic lymphocytic leukemia, small lymphocytic lymphoma, mantle cell lymphoma, Waldenström macroglobulinemia, and marginal zone lymphoma [4]. Here, we present the case of a young female who developed severe left ventricular systolic dysfunction after four months of therapy with ibrutinib. However, the systolic function recovered completely once ibrutinib was stopped.

## Case presentation

A 42-year-old female with a medical history significant for lymphoplasmacytic lymphoma (diagnosed via bone marrow and lymph node biopsy), iron deficiency anemia requiring transfusion, bronchial asthma, chronic kidney disease stage G1 A3, and chronic venous insufficiency status post-bilateral endovenous laser ablation of saphenous veins. Her previous malignancy work showed low-grade plasmacytoid lymphocytic lymphoma with elevated free light-chain levels. She was started on rituximab and Ibrutinib. Ibrutinib (420 mg) was started four months before the admission (in June 2021). It was stopped for a short duration because of gum bleeding, was restarted at a lower dose (140 mg), and continued until the presentation (in October 2021).

**Figure 1:**
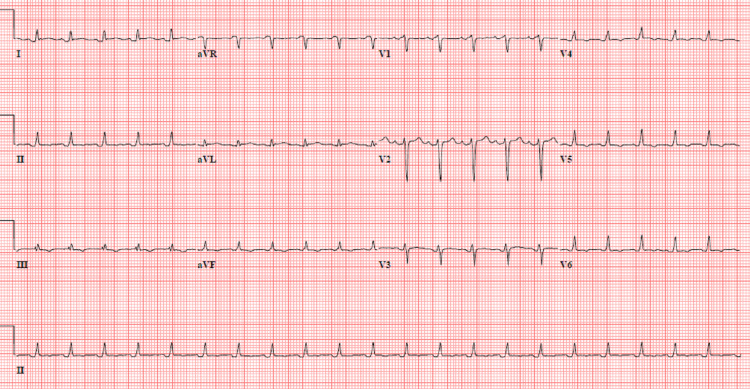
Electrocardiogram showing sinus tachycardia and non-specific T-wave changes.

The patient presented to the emergency department with right upper quadrant pain associated with fever, chills, and weakness of two-day duration. Her vitals in the emergency department were temperature of 97.9°F, heart rate of 136 beats/minute, blood pressure of 74/34 mmHg, and O_2_ saturation of 96% on room air. An electrocardiogram showed sinus tachycardia (Figure 1). On examination, she was alert and oriented with right upper quadrant abdominal tenderness, bilateral pitting pedal edema, and normal vesicular breath sounds bilaterally.

Initial laboratory findings were as following: hemoglobin, 7.8 g/dL (12-16 g/dL), white blood cells, 5.2 k/µL (4.8-10.8 k/µL), neutrophil, 90.1%, platelets, 163 k/µL (150-400 k/µL), lactic acid, 2.9 mmol/L (0.5-1.6 mmol/L), lipase, 5 U/L (≤61 U/L), serum albumin, 3.8 g/dL (3.2-4.8 g/dL), total bilirubin, 1.7 mg/dL (0.2-1.2 mg/dL), alkaline phosphatase, 131 U/L (42-98 U/L), blood urea nitrogen, 23 mg/dL (6-20 mg/dL), serum creatinine, 1.5 mg/dL (0.5-1.5 mg/dL), pro-brain natriuretic peptide (pro-BNP): 70,000 pg/mL (0-125 pg/mL), alanine aminotransferase (ALT), 10 U/L (5-40 U/L), and aspartate transaminases (AST), 21 U/L (9-36 U/L).

**Figure 2:**
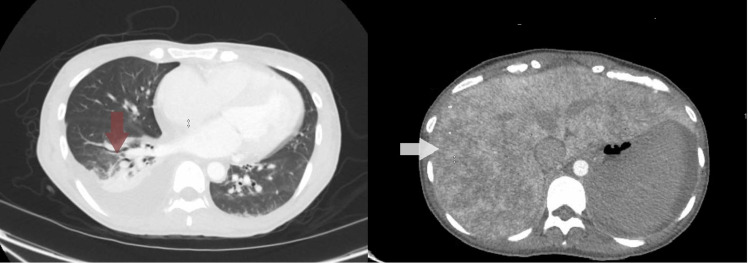
Computerized tomography scan of the abdomen and pelvis showing right-sided effusion (red arrow) and nutmeg liver (white arrow).

Because of hypotension, vasopressors were started after a fluid challenge. Broad-spectrum intravenous antibiotics, including vancomycin, piperacillin-tazobactam, and metronidazole, were initiated. Pan-cultures (blood culture, urine culture, and respiratory culture) were sent. The chest radiograph demonstrated right lower lobe opacities. A computerized tomography scan of the abdomen and pelvis with intravenous contrast showed moderate right-sided pleural effusion with right lower lobe consolidation and mottled liver enhancement compatible with nutmeg liver (Figure 2). Ultrasound of the abdomen showed a patent portal vein with mild pelvic ascites and hepatomegaly.

The patient was admitted to the intensive care unit and vasopressors were continued. Blood culture grew *Escherichia coli*, while urine culture, respiratory culture, and pneumonia workup were negative. She developed acute respiratory failure requiring non-invasive positive pressure ventilation from which she was weaned off eventually. She was found to have ileus with colonic loop dilatation on X-ray of the kidney, ureter, and bladder (KUB), which was managed conservatively. Her clinical course was complicated by worsening metabolic acidosis with acute kidney injury, anasarca, oliguria, worsening pericardial, and bilateral pleural effusions.

The cause of renal failure was multifactorial, contributed by sepsis, hypotension, and contrast administration. Nephrology was consulted, and she was initiated on hemodialysis. She underwent thoracentesis of the right-sided pleural effusion, and analysis showed exudative effusion with negative gram stain and cultures. Vasopressors were tapered off as her blood pressure slowly improved.

**Figure 3:**
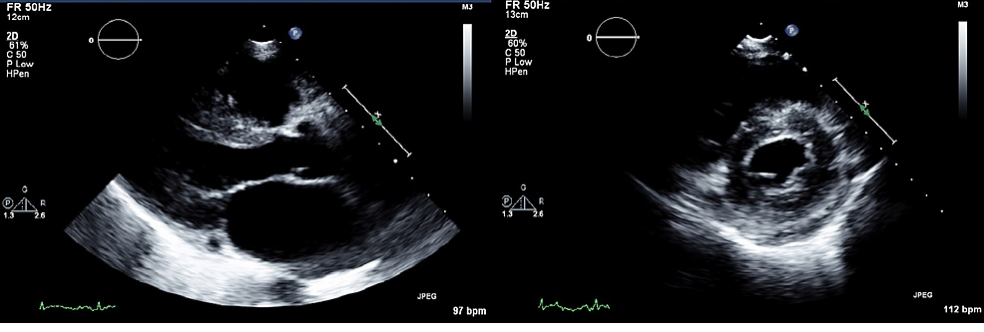
Left side: Parasternal long-axis view of the echocardiogram at baseline showing non-dilated left ventricle; right side: parasternal short-axis view at papillary muscle level showing non-dilated left ventricle (April 2021).

Before the current presentation, her echocardiogram done a few months back (in April 2021, before initiation of ibrutinib therapy) showed a normal left ventricular ejection fraction of 62% (Figure 3). In addition, a stress echocardiogram done approximately around the same time was also negative for any inducible ischemia.

**Figure 4:**
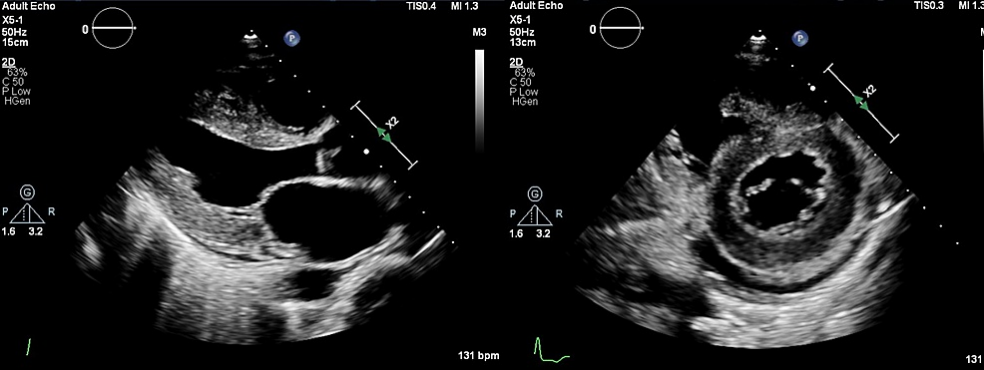
Left side: Parasternal long-axis view of the echocardiogram showing the dilated left atrium and left ventricle; right side: parasternal short-axis view at papillary muscle level showing dilated left ventricle and minimal pericardial effusion (October 20, 2021).

An echocardiogram obtained during this admission showed new-onset systolic cardiac dysfunction with an ejection fraction of 36% (Figure 4). This change could be associated with initiating Ibrutinib therapy four months before admission.

**Figure 5:**
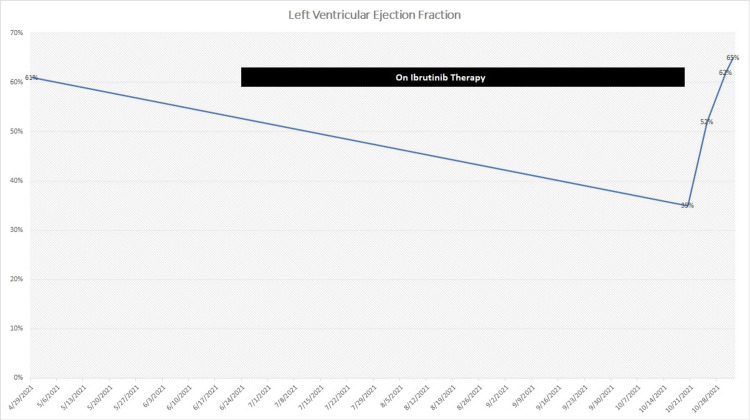
Drop and subsequent improvement in left ventricular ejection fraction with initiation versus discontinuation of ibrutinib therapy.

The differentials considered for cardiac dysfunction were septic cardiomyopathy versus drug-induced cardiomyopathy secondary to ibrutinib. However, the presence of nutmeg liver pointed toward the chronicity of dysfunction, and hence, Ibrutinib was stopped. Sequential transthoracic echocardiograms were obtained during hospitalization, which demonstrated improving ejection fraction (Figure 5).

As her hemodynamic status improved, she was started on a low-dose beta-blocker. Her pericardial effusion reduced in size with continued hemodialysis sessions. However, her renal function did not recover. She was discharged with outpatient dialysis and follow-up with oncology and cardiology.

## Discussion

Cardiotoxicity due to chemotherapeutic drugs has been an evolving field, with a lot of interest generated lately due to the introduction of a large number of chemotherapeutic agents such as monoclonal antibodies and small-molecule inhibitors. While some of these agents affect the heart directly by causing vasospasm (5-fluorouracil) [5], free radical injury (anthracyclines), or intracellular signaling pathway alteration (sunitinib, trastuzumab), others can affect indirectly via the development of hypertension (bevacizumab, ibrutinib) and arrhythmias [6,7]. In contradiction to monoclonal antibodies (ending with “mab”) such as trastuzumab which affect the intracellular signaling pathways by attaching outside of the cells, the small-molecule inhibitors (ending with “nib”) affect these pathways by reaching into the cell and altering the interplay of molecules. These molecules can produce both “on target” and “off-target” effects.

A new class of tyrosine kinase inhibitors (TKIs) called BTKIs was introduced as they were very effective against B-cell lymphocytes. Ibrutinib was introduced as the first agent in this class, followed by acalabrutinib and zanubrutinib. They act by attaching to the ATP-binding domain of BTK. BTK acts by activating phospholipase C γ2 (PLCγ2), which, in turn, stimulates protein kinase C, thus activating a series of intracellular signaling that leads to B-cell proliferation. Inhibition of BTK causes downregulation of these downstream pathways and brings out B-cell-inhibiting effects. However, it also acts on 50 other tyrosine kinases in the body, contributing to many off-target effects seen with ibrutinib [8]. It has been known to cause hypertension and atrial fibrillation (8%) in many patients [9]. Hypertension has been seen to occur after almost a year of Ibrutinib use, while the temporal correlation with atrial fibrillation and cardiomyopathy is not reported in the literature. Depletion of intracellular PI3/anaplastic lymphoma kinase and development of atrial fibrosis have been proposed to be the possible explanation for the occurrence of atrial fibrillation [10].

The common cardiovascular side effects of ibrutinib therapy include hypertension (14-19%) [11], peripheral edema, atrial fibrillation (≤8%), atrial flutter (≤8%), ventricular arrhythmias including ventricular premature contractions, ventricular tachycardia, and ventricular fibrillation [12]. While atrial fibrillation appears to be relatively common (in fact, it was the most common cause of drug discontinuation in one retrospective study of patients with chronic lymphocytic leukemia [13]), systolic heart failure in the absence of arrhythmia is a relatively rare finding. Index patient developed reversible left ventricular global hypokinesia detected after four months of ibrutinib use, which started improving within a week of stopping the drug.

The elimination half-life of ibrutinib is four to six hours [14]. Some drugs, such as adriamycin, can cause long-term irreversible cardiac dysfunction, while others, such as trastuzumab, cause predominantly reversible cardiac dysfunction [6]. Limited case reports of cardiomyopathy attributed to ibrutinib have described both reversible and partially reversible cardiac dysfunction [4,15-17]. Although the development of hypertension and atrial arrhythmias leading to cardiomyopathy can occur, our case is unique in that both hypertension and atrial fibrillation were not found. Moreover, it is essential to consider ischemia as a cause in such cases. A reported normal stress echocardiogram is done two months before starting ibrutinib, which strongly argues against ischemia as the etiology of cardiac dysfunction in this case. This leads authors to propose a direct cellular effect of Ibrutinib on cardiac myocyte leading to cardiac dysfunction as the possible mechanism. Alteration of “off-target” intracellular signaling pathways, depletion of intracellular ATPs, the affliction of mitochondrial metabolism can be the potential factors causing cardiac dysfunction in our case [8]. Elevated ProBNP has been linked to TKIs [18]. Our patient had significantly elevated ProBNP levels. Moreover, she had pericardial effusion, but it can be explained by renal dysfunction, which developed in the patient.

In critically ill patients with multiorgan dysfunction and sepsis, it can be a real challenge to tease out different causes of cardiac dysfunction, such as septic cardiomyopathy. However, a strong temporal correlation of development of cardiac dysfunction with ibrutinib and return of cardiac function toward normal with stoppage of the drug strongly favors ibrutinib-induced reversible cardiotoxicity.

In the future, with the development of more specific BTKIs, the cardiotoxic effects are likely to decrease, and study of the side effect profile of those agents can shed more light on the mechanism of cardiotoxicity in these patients. In addition, screening for the development of cardiac dysfunction with longitudinal strain imaging on echocardiograms can be proposed to screen for the development of such off-target cardiac effects.

## Conclusions

Ibrutinib is a chemotherapeutic agent and an irreversible inhibitor of Bruton tyrosine kinase. It has been associated with significant cardiotoxic effects, the most common being hypertension and atrial fibrillation. Non-ischemic cardiomyopathy is a rare complication of ibrutinib, especially in the absence of atrial fibrillation. The clinicians should watch out for the signs and symptoms of heart failure in patients who are started on ibrutinib chemotherapy. A thorough evaluation of patient history, routine surveillance, and symptoms assessment should be done while on this therapy. The compromised myocardial function appears to be transient and usually improves after the drug is stopped.

## References

[1] Herman SE, Gordon AL, Hertlein E (2011). Bruton tyrosine kinase represents a promising therapeutic target for treatment of chronic lymphocytic leukemia and is effectively targeted by PCI-32765. Blood.

[2] Cinar M, Hamedani F, Mo Z, Cinar B, Amin HM, Alkan S (2013). Bruton tyrosine kinase is commonly overexpressed in mantle cell lymphoma and its attenuation by Ibrutinib induces apoptosis. Leuk Res.

[3] Lee CS, Rattu MA, Kim SS (2016). A review of a novel, Bruton's tyrosine kinase inhibitor, ibrutinib. J Oncol Pharm Pract.

[4] Kyi HH, Zayed Y, Al Hadidi S (2019). Ibrutinib-induced cardiomyopathy. J Community Hosp Intern Med Perspect.

[5] Yildirim M, Parlak C, Sezer C, Eryilmaz R, Kaya C, Yildiz M (2011). Coronary vasospasm secondary to 5-fluorouracil and its management: case report. Eurasian J Med.

[6] Henning RJ, Harbison RD (2017). Cardio-oncology: cardiovascular complications of cancer therapy. Future Cardiol.

[7] Higgins AY, O'Halloran TD, Chang JD (2015). Chemotherapy-induced cardiomyopathy. Heart Fail Rev.

[8] Brown SA, Ray JC, Herrmann J (2020). Precision cardio-oncology: a systems-based perspective on cardiotoxicity of tyrosine kinase inhibitors and immune checkpoint inhibitors. J Cardiovasc Transl Res.

[9] Burger JA, Tedeschi A, Barr PM (2015). Ibrutinib as initial therapy for patients with chronic lymphocytic leukemia. N Engl J Med.

[10] Sestier M, Hillis C, Fraser G, Leong D (2021). Bruton's tyrosine kinase inhibitors and cardiotoxicity: more than just atrial fibrillation. Curr Oncol Rep.

[11] Dickerson T, Wiczer T, Waller A (2019). Hypertension and incident cardiovascular events following ibrutinib initiation. Blood.

[12] Salem JE, Manouchehri A, Bretagne M (2019). Cardiovascular toxicities associated with ibrutinib. J Am Coll Cardiol.

[13] Mato AR, Nabhan C, Barr PM (2016). Outcomes of CLL patients treated with sequential kinase inhibitor therapy: a real world experience. Blood.

[14] Brunton LL, Hilal-Dandan R, Knollmann BC (2018). Goodman & Gilman's: the pharmacological basis of therapeutics. https://accessmedicine.mhmedical.com/book.aspx?bookID=2189.

[15] Liang SH, Chiu CF, Bai LY (2019). Ibrutinib-associated reversible cardiomyopathy. J Oncol Pract.

[16] Wasserstrum Y, Raanani P, Kornowski R, Iakobishvili Z (2016). Concomitant treatment with ibrutinib and amiodarone causing reversible heart failure syndrome. Isr Med Assoc J.

[17] Wallace N, Wong E, Cooper D, Chao H (2016). A case of new-onset cardiomyopathy and ventricular tachycardia in a patient receiving ibrutinib for relapsed mantle cell lymphoma. Clin Case Rep.

[18] Witteles RM (2016). Biomarkers as predictors of cardiac toxicity from targeted cancer therapies. J Card Fail.

